# 응급실 간호사 직무스트레스와 수면, 신체활동, 전문직 정체성, 동료지지가 직무만족도에 미치는 영향: 로이 적응이론 기반 횡단적 기술 연구

**DOI:** 10.7586/jkbns.26.019

**Published:** 2026-05-27

**Authors:** Min Young Kim, Hyun Jin Chung, Minhee Suh

**Affiliations:** 1 Department of Nursing, Inha University Hospital, Incheon, Korea; 1 인하대학교병원 간호본부; 2 School of Nursing, Inha University, Incheon, Korea; 2 인하대학교 간호대학

**Keywords:** Emergency nursing, Job satisfaction, Occupational stress, Sleep, Social identification, 응급 간호, 직무 만족도, 직무 스트레스, 수면, 전문직 정체성

## Abstract

**Purpose:**

This study investigated factors influencing job satisfaction among emergency department (ED) nurses, focusing on job stress, sleep, physical activity, professional identity, and peer support, based on Roy’s adaptation model.

**Methods:**

This descriptive cross-sectional study included 64 nurses working at a regional emergency medical center. Job stress and job satisfaction were measured using Kim and Gu’s nursing job stress questionnaire and Lee’s job satisfaction questionnaire, respectively. Sleep and physical activity were objectively measured using a wrist-worn actigraph for seven consecutive days. Professional identity and peer support were assessed using self-report questionnaires.

**Results:**

The mean job satisfaction score was 117.70. Job satisfaction showed significant positive correlations with total sleep time (*p* = .024), daily step count (*p* = .032), professional identity (*p* < .001), and peer support (*p* < .001). Hierarchical regression analyses showed that the final model explained 65.0% of the variance in job satisfaction (*p* < .001). Total sleep time (*p* = .028), professional identity (*p* < .001), and peer support (*p* < .001) were significant predictors. However, job stress and physical activity were not significant predictors in the final model.

**Conclusion:**

Professional identity was the strongest predictor of job satisfaction among ED nurses. Job satisfaction among ED nurses appeared to be more closely associated with total sleep time than with sleep quality. These findings suggest that improving job satisfaction requires not only managing physiological factors, such as adequate sleep duration, but also strengthening professional identity and fostering supportive peer relationships.

## 서론

### 1. 연구의 필요성

응급실 이용률이 지속적으로 높아지면서 응급실 과밀화 문제가 꾸준히 제기되고 있으며 2020년 이후 코로나바이러스 감염증으로 응급실 이용율은 더욱 증가하였다[1]. 이러한 상황에서 응급실 간호사는 환자, 보호자와의 복잡한 인간관계 및 언어적, 신체적 폭력에 노출되어 타 부서 간호사 보다 직무스트레스 수준이 높은 것으로 알려져 있으며[2], 이는 간호의 질이나 직무만족도에 영향을 미칠 수 있다[3,4].

직무만족도란 직무 자체를 즐겁고 긍정적인 것으로 인식하는 직무에 대한 태도로서, 간호사의 이직과 가장 관련 있는 것으로 보고되고 있으며[5] 간호사의 직무만족도는 결근율, 이직률, 업무 생산성에 직접적인 영향을 주어[6,7] 간호조직의 간호의 질과 비용 효과 증진 및 조직 목표 달성에 매우 중요한 요인이다.

직무스트레스는 간호사의 직무만족도에 영향을 미치는 요인으로 알려져 있으며[8], 스트레스는 3교대 업무 특성과 함께 간호사의 수면이나 신체활동을 변화시킬 수 있음을 고려할때[9,10], 장기간의 직무스트레스 및 교란된 수면과 신체활동은 직무만족도와 관련될 수 있다[11]. 그럼에도 불구하고 직무만족도와 관련되는 요인으로서의 직무스트레스, 수면 및 신체활동을 조사한 연구는 부족한 실정이다. 따라서, 직무스트레스에 대한 응급실 간호사들의 수면과 신체활동을 조사하고 이러한 생행동적 변화와 직무만족도와의 관련성을 조사하여, 스트레스 수준이 특히 높은 응급실 간호사의 직무스트레스 관리와 조절 측면에 초점을 두고 다양한 직무만족도 증진 요인을 심도 있게 분석한 연구가 필요하다. 뿐만 아니라, 불규칙한 수면이나 신체활동 양상은 회상에 의한 설문조사 방법으로는 측정하는데 한계가 있을 수 있으므로, 최신 센싱기술을 이용한 액티그래프(actigraphy)를 활용하여 측정하여 보다 객관적으로 측정하는 것이 필요하다.

이외에도 전문직 정체성 역시 직무만족도와 상관성을 보이는 것으로 알려져 있고[12], 사회적 지지와 같은 주변의 지원[13] 또한 직무만족도와 관련이 있는 것으로 보고된 바 있다. 그러나 직무만족도에 관련되는 개별 요인들에 대한 단편적 연구들이 주를 이루고 있으며, 직무만족도 관련 요인에 대한 통합적인 연구는 미흡한 실정이다.

Roy의 적응 이론(Roy’s adaptation model)에 의하면 인간은 적응체계로서, 삶을 유지하기 위하여 환경에서 받은 자극을 다양한 적응 양식(생리적 양식, 자아개념 양식, 역할기능 양식, 상호의존 양식)을 통해 조절하고, 그 결과 적응적 반응 또는 부적응적 반응을 나타낸다고 하였다[14]. 수면의 변화는 스트레스에 대한 적응 방식의 일종일 수 있으므로[15] 직무스트레스 자극에 대한 생리적 적응 양식으로서 응급실 간호사들의 수면과 신체활동 양상을 확인할 수 있을 것이다. 전문직 정체성과 동료 및 주변의 지지 역시 스트레스와 관련이 있으므로[16,17], 이 같은 요인들을 직무스트레스에 대한 적응양식으로서 자아개념 및 역할기능 양식, 상호의존 양식으로 조사하여 반응 으로서의 직무만족도와 관련성을 확인해 볼 수 있을 것으로 보인다. 이를 통해 응급실 간호사의 직무스트레스와 다양한 적응 양식의 측면을 포함하고, 이러한 요인들이 직무스트레스와 직무만족도 사이에서 어떠한 역할을 하는지에 대해 통합적으로 살펴볼 수 있을 것으로 생각된다.

이에 본 연구에서는 응급실 간호사들의 직무스트레스와 수면이나 신체활동이 직무만족도와 어떻게 관련되는지 조사하고, 로이의 적응이론에 기반하여 수면이나 신체활동 같은 생리적 양식과 전문직 정체성(자아개념 및 역할기능 양식), 동료 지지(상호의존 양식)가 반응으로써 직무만족도에 미치는 영향을 확인하고자 한다.

### 2. 연구의 목적

본 연구의 목적은 응급실간호사를 대상으로 직무스트레스, 수면, 신체활동, 전문직 정체성, 동료지지가 직무만족도에 어떤 영향을 미치는지를 파악하고자 한다. 수면, 신체활동을 객관적으로 측정하기 위해 최신 센싱 기술인 액티그래프를 이용하여 측정하고, 전문직 정체성, 동료지지는 설문지를 이용하여 측정한 후, 각각의 변수들이 직무만족도에 영향을 미치는지 확인하고자 한다.

1) 응급실간호사의 직무스트레스, 전문직 정체성, 동료지지, 직무만족도를 조사한다.

2) 응급실간호사의 수면, 신체활동을 액티그래프를 사용하여 측정한다.

3) 응급실간호사의 직무스트레스, 수면, 신체활동, 전문직 정체성, 동료지지와 직무만족도의 관련성을 분석한다.

4) 응급실간호사의 직무스트레스, 수면, 신체활동, 전문직 정체성, 동료지지가 직무만족도에 영향을 미치는지 분석한다.

### 3. 이론적 기틀

본 연구의 이론적 기틀은 Roy의 적응 이론을 기반으로 하고 있다[18]. 인간은 환경으로부터 받은 자극을 조절 기전을 통해 조절하고, 적응 양식을 거쳐, 결과적으로 적응적 또는 비적응적 반응을 하게 된다는 것이 Roy의 적응 이론의 핵심이다. 인간의 적응체계에 투입되는 자극은 초점 자극으로, 일반적으로 스트레스가 초점 자극에 해당될 수 있으며, 인간은 스트레스 자극을 처리하기 위해서 에너지를 소모한다. 본 연구에서는 직무스트레스를 초점 자극으로 보았다.

인간이 받은 자극은 자극에 대한 기본 반응인 조절 기전을 통해 조절된다. 조절 기전으로는 인체의 신경적, 화학적 대처인 조절기 하위체계와 인지/정보과정, 학습, 판단, 정서 과정을 통해 반응하는 인지기 하위체계가 있다. 그러나 이러한 조절 기전은 인간의 내적인 자동적 조절 과정으로 개념화되어 있으며, 조절 기전과 직무만족도 간의 관련성을 조사한 선행 연구가 없어 본 연구에서는 포함하지 않았다.

조절된 자극은 다양한 적응 양식 유형으로 적응 과정을 거치게 된다. Roy 적응 이론[18]에서는 인간의 적응양식으로 생리적 양식, 자아개념 양식, 역할기능 양식, 상호의존 양식을 제시하였다. 생리적 양식은 자극에 대한 생리적 통합성의 욕구를 말하며 휴식 및 활동과 관련된 적응이 포함된다. 본 연구에서는 수면 상태와 신체활동을 생리적 적응 양식으로 보았다. 자아개념 양식은 자신에 대한 감정이나 신념을 통합한 요인을 말하며 역할기능은 사회에서 주어진 지위에 따른 의무를 이행하는 사회적 통합을 의미한다. 본 연구에서는 전문직 정체성을 자아개념 양식 및 역할기능 양식으로 보았다. 적응 양식 중, 상호의존 양식은 가치, 존경, 사랑 등을 서로 주고받으면서 타인과의 상호의존 욕구를 충족시켜주는 양식을 말하며, 본 연구에서는 상호의존 양식은 동료 지지를 말한다.

**Figure 1.**
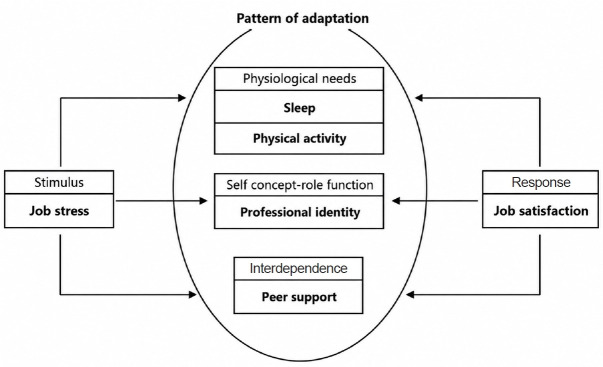
Theoretical model of the study.

본 연구에서는 Roy의 적응 이론의 결과 지표인 반응을 직무 만족도로 측정하였다. Roy의 적응 이론에 기반하여 본 연구의 개념을 설명하면, 간호사가 받은 자극인 직무스트레스는 조절 기전으로 조절이 된 후에 적응 양식 중 생리적 양식인 수면 행동이나 신체활동, 자아개념 양식 및 역할기능 양식인 전문직 정체성, 상호의존 양식인 동료 지지를 거쳐서 반응인 직무 만족도로 표출된다. 이를 바탕으로 하여 설정한 본 연구의 이론적 기틀은 Figure 1과 같다.

## 연구 방법

### 1. 연구 설계

본 연구는 로이의 적응이론을 기반으로 권역응급의료센터 응급실간호사의 직무스트레스 및 수면, 신체활동, 전문직 정체성, 동료지지를 조사하고, 이러한 스트레스와 적응양상이 직무 만족도에 미치는 영향 및 관계를 파악하고자 하는 서술적 조사연구이다. 본 연구는 관찰연구 보고지침인 STROBE (Strengthening the Reporting of Observational Studies in Epidemiology) statement에 근거하여 연구 내용을 보고하였다.

### 2. 연구 대상

연구대상은 응급실에 근무 중인 간호사 중 적어도 3개월 이상 근무하였으며, 연구 목적과 방법을 이해하고 자발적으로 연구에 참여한 자로, 3교대 근무를 하지 않거나 현재 수면제 복용 등의 의도적으로 수면을 조절하고 있는 자는 제외하였다.

본 연구 대상자의 표본 수는 G*power 3.1 program (Heinrich-Heine-Universität, Düsseldorf, German)을 활용하여 산정하였다. 직무 만족도에 영향을 미치는 요인을 파악하기 위해 회귀분석(multiple linear regression) 통계를 기준으로 검정력 .90, 큰 효과크기 .35, 유의수준 .05, 변수 수 13개를 기준으로 설정하였을때 최소 표본수 64명이었고, 탈락한 사람은 없었으며, 최종분석에 64명이 포함되었다.

### 3. 연구 도구

#### 1) 일반적 특성

일반적 특성으로 성별, 연령, 학력, 거주형태, 배우자 유무, 임상경력, 월급, 밤 근무 유무를 포함하였다.

#### 2) 직무 스트레스

Kim과 Gu[19]가 개발한 축약형 간호사 직무스트레스 측정도구를 사용하여 평가하였다. 본 도구는 업무 환경에 의한 압박으로서의 직무스트레스 수준을 파악하기 위한 총 16문항의 도구로, 업무량 및 업무환경 5문항, 역할갈등 8문항, 업무의 책임 3문항으로 구성되어 있고, 각 문항은 5점 Likert 척도로 평가한다. 점수가 높을수록 간호사가 지각하는 직무스트레스가 높음을 의미한다. Kim과 Gu [19]는 도구의 신뢰도를 Cronbach’s *α* = .94, Yoo 등[20]은 Cronbach’s *α* = .89 로 보고하였다. 본 연구에서의 신뢰도는 Cronbach’s *α* = .87이었다.

#### 3) 수면, 신체활동 측정

수면과 신체활동은 웨어러블 액티그래프 기기인 액티그래프 GT3X (Ametris, Pensacola, Florida, USA)를 이용하여 측정하였다. 이 기기는 3개의 축에서 신체의 움직임을 측정하여 자세 및 활동량 정보, 수면상태를 모니터링 한다. 액티그래프는 대상자의 주로 쓰지 않는 측의 손목에 7일간 착용하게 하여 측정하였으며, 신체 움직임은 30 Hz에서 1분 간격으로 감지하도록 설정되었다. 이 기기를 이용한 수면 및 신체활동 측정의 신뢰도는 비교적 안정적인 것으로 보고되었다[21].

액티그래프로 측정된 자료는 ActiLife 6.13.4 software (Ametris, Pensacola, Florida, USA)를 이용하여 분석하였다. Choi의 알고리즘[22]을 이용하여 착용시간을 추출하였고, 측정한 기간 동안의 일 평균치를 산출하여 분석에 이용하였다. 수면지표로는 수면효율(sleep efficiency), 총 수면시간(time in bed, TIB), 실제 총 수면시간(total sleep time, TST), 수면 후 각성시간(wake after sleep onset, WASO), 야간 평균 각성 횟수(wake episode, WE), 야간 1회 평균 각성시간(mean wake episode, MWE)을 산츨하였다. 신체활동 지표는 일 평균 걸음 수(daily step count)와 신체활동 강도(정적 활동 및 저강도 활동, 중강도 활동, 고강도 활동의 일 평균 시간)를 분석하였다. 기기에서 측정된 측정 단위(counts per minute, CPM)에 Freedson adult 알고리즘을 적용하여[23] 0~99 CPM은 정적 활동, 100~1,951 CPM은 저강도의 활동, 1,952~5,724 CPM은 중강도의 활동, 5,725 CPM 이상의 활동은 고강도 활동으로 분류하였다.

#### 4) 전문직 정체성

Hall과 Snizek의 전문직 정체성 도구를 Kim이 수정, 보완한 도구[24]를 사용하여 평가하였다. 본 도구는 총 17문항으로, 자율성 5문항, 서비스 2문항, 소명의식 6문항, 전문조직활동 4문항으로 구성되었으며, 5점 Likert 척도로서 총 점수가 높을수록 전문직 정체성이 높은 것을 의미한다. 도구의 신뢰도는 Kim[24]은 Cronbach’s *α* = .75, Yoo 등[20]은 Cronbach’s *α* = .88로 보고하였다. 본 연구에서 신뢰도는 Cronbach’s *α* = .83이었다.

#### 5) 동료지지

Peter 등이 개발하고 Kim이 번안한 도구[25]를 사용하여 평가하였다. 본 도구는 상사의 지지 4문항, 동료의 지지 4문항의 2개 하위 영역 총 8문항을 포함하며, 각 문항은 Likert 5점 척도이다. 점수가 높을수록 간호사가 지각하는 동료 지지가 높음을 의미한다. Kim[25]은 도구의 신뢰도를 Cronbach’s *α* = .79로 보고하였으며, 본 연구에서 신뢰도는 Cronbach’s *α* = .85이었다.

#### 6) 직무 만족도

Lee 등[26]이 개발한 간호사의 직무만족 측정도구를 사용하여 평가하였다. 본 도구는 총 33문항으로, 조직의 인정 및 전문적 성취 영역 9문항, 간호 전문직을 위한 인간적 성숙 영역 6문항, 존중과 인정의 인간관계 영역 8문항, 간호사로서의 책무완수 영역 4문항, 직업의 안정성과 보람 영역 3문항, 전문적 역량 발휘 영역 3문항을 포함하며, 각 문항은 5점 Likert 척도로 평가한다. 점수가 높을수록 간호사의 직무만족도가 높음을 의미한다. Lee 등의 연구[26]에서의 도구의 신뢰도는 Cronbach’s *α* = .95, 본 연구에서의 신뢰도는 Cronbach’s *α* = .92이었다.

### 4. 자료 수집

연구 대상자는 인하대학교병원 권역응급센터내 간호사 게시판에 ‘대상자 모집 공고문’을 부착하여 모집하여 자발적인 연구 참여 의사를 밝힌 대상자에게 연구 설명문을 제공하고 서면동의를 받은 후, 액티그래프 착용 방법을 교육하였다. 대상자는 주로 사용하지 않는 손목에 액티그래프를 7일 동안 착용하였으며, 기기 반납 시 자가보고형 설문지를 작성하였다. 이후 연구자는 액티그래프와 설문지를 수거하였다.

### 5. 자료 분석

본 연구의 수집된 자료는 SPSS WIN 28.0 (IBM Corp., Armonk, NY, USA) 프로그램을 사용하여 통계적으로 분석하였다. 연구 변수들의 정규성을 확인하기 위해 Shapiro-Wilk 검정을 시행한 후, 정규성을 만족하지 않는 변수들은 로그 변환하여 분석하였으며 구체적인 방법은 다음과 같다.

1. 대상자의 인구통계학적 특성에 대한 특성을 파악하기 위하여 빈도분석을 실시하였고, 직무만족도와 관련성을 살펴보기 위하여 Independent t-test 또는 one-way ANOVA로 분석하였다.

2. 주요 변수인 직무스트레스, 수면, 신체활동, 전문직 정체성, 동료지지 및 종속변수인 직무만족도에 대해 평균과 표준편차 및 중앙값과 사분위 범위를 제시하였다.

3. 직무스트레스, 수면 및 신체활동 지표, 전문직 정체성, 동료지지와 직무만족도 간의 관련성을 분석하기 위해 Pearson 상관분석을 이용하여 분석하였다.

4. 직무만족도에 관련되는 요인을 확인하기 위해 성별, 직무스트레스, 수면 및 신체활동, 전문직 정체성, 동료지지를 독립변수로 투입하여 위계적 회귀분석을 실시하였다.

### 6. 윤리적 고려

본 연구는 연구자가 소속한 인하대학교병원의 연구윤리심의위원회 승인을 받았다. (IRB No. 2024-01-001) 자료 수집 전 연구의 목적과 방법을 이해하고 이에 대해 훈련 받은 연구자가 연구 대상자에게 연구의 목적과 방법 등에 대해서 충분히 설명하였다. 사전 동의서를 통해 연구 설문 참여에 자발적으로 동의한 대상자에게만 설문지 배부 및 액티그래프를 착용하였으며, 액티그래프와 설문 조사를 통해 얻어진 자료는 연구 목적 이외에는 사용하지 않으며 대상자가 원하면 언제든지 철회할 수 있음을 알렸다.

## 연구 결과

### 1. 연구대상자의 특성과 직무 만족도

본 연구에 참여한 대상자는 총 64명으로, 연령은 26~29세가 33명(51.6%)으로 가장 많았으며, 성별은 여성이 56명(87.5%)으로 대부분을 차지하였다(Table 1). 학력은 대학교 졸업이 58명(90.6%)이었고, 거주형태는 가족 또는 친구와 함께 거주하는 경우와 1인 가구가 각각 32명(50.0%)으로 동일하게 나타났으며, 미혼이 55명(85.9%)이었다. 임상경력은 3년 미만이 31명(48.4%)으로 가장 많았고, 월 소득은 월 300~400만원 구간이 63명(98.4%)으로 대부분이었다. 액티그래프를 착용한 기간 중 밤근무가 포함되어 있었던 대상자는 16명(25.0%)이었다.

대상자의 일반적 특성에 따른 직무만족도 차이를 분석한 결과, 성별에 따라 유의미한 차이가 나타났다(*p* = .004). 남성이 여성보다 직무만족도가 유의하게 높은 것으로 나타났다. 그러나 연령, 학력, 거주형태, 배우자 유무, 임상경력, 월급, 밤 근무 유무에 따른 직무만족도에서는 통계적으로 유의한 차이가 없었다.

### 2. 주요 연구변수의 기술통계

대상자의 주요 변수에 대한 기술통계 분석 결과는 Table 2에 제시되어 있다. 먼저 직무스트레스는 평균 61.48 ± 8.70점, 직무만족도는 평균 117.70 ± 13.38점으로 나타났다.

수면 관련 지표에서는 수면 효율(efficiency)이 평균 85.88 ± 4.53%, 총 침대 머문 시간(TIB)은 465.84 ± 106.29분, 총 수면 시간(TST)은 404.43 ± 104.47분, 수면 중 깨어 있는 시간(WASO)은 59.81 ± 17.91분이었다. 수면 중 깨어 있는 에피소드 횟수(WE)는 19.45 ± 5.40회, 평균 깨어 있는 에피소드 시간(MWE)은 4.07 ± 2.79분으로 측정되었다.

신체활동 관련 지표에서는 신체활동 강도(metabolic equivalents of task, METs)가 평균 1.37 ± 0.17 METs, 일일 걸음 수(daily step count)는 9,723.84 ± 2,252.90보로 나타났다. 하루 평균 좌식 활동 시간(sedentary)은 641.34 ± 124.30분, 저강도 활동 시간(light)은 430.48 ± 76.08분, 중강도 활동 시간(moderate)은 192.13 ± 47.36분, 고강도 활동 시간(vigorous)은 0.00 ± 0.00분이었으며, 강도별 활동시간의 비율은 각각 50.19%, 34.51%, 15.30%, 0%이었다.

전문직 정체성은 평균 63.70 ± 6.77점, 동료지지는 평균 32.61 ± 3.97점이었다.

### 3. 연구 변수 간의 상관관계

주요 변수 간 상과관계 분석 결과는 다음과 같다(Table 3). 수면 관련 지표인 실제 총 수면 시간(TST)과 직무만족도는 정적 상관성을 보여, 총 수면시간이 길수록 직무만족도가 높았다(*p* < .050). 그러나 수면 효율성(efficiency)과 직무만족도 간의 상관성은 유의하지 않는 것으로 나타났다.

일일 걸음 수는 직무만족도와 유의한 정적 상관관계를 나타내어(*p* < .050), 일일 걸음 수가 많을수록 직무만족도가 증가하는 경향을 보였다. 반면, 강도별 신체활동은 직무만족도와 통계적으로 유의한 상관관계를 보이지 않았다.

전문직 정체성과 직무만족도는 정적 상관성을 보여, 전문직 정체성이 높을수록 직무만족도가 유의하게 증가함을 확인할 수 있었다(*p* < .001). 동료지지와 직무만족도도 정적 상관성을 나타내, 동료지지가 높을수록 직무만족도가 증가함을 알 수 있었다(*p* < .001).

직무스트레스와 직무만족도 간에도 유의미한 상관관계를 보이지 않았다.

### 4. 직무만족도에 영향을 미치는 요인

직무만족도에 영향을 미치는 요인을 살펴보기 위해 위계적 회귀분석을 실시하였다(Table 4). 다중회귀분석에 앞서 독립변수 간 다중공선성을 검토하기 위해 공차한계(tolerance)와 분산팽창지수(variance inflation factor, VIF)를 확인한 결과, 모든 변수의 공차한계가 0.69~0.94로 0.1 이상이었고, VIF가 1.07~1.45로 10 이하의 수치로 나타나 다중공선성의 문제는 없었다. 또한 Durbin–Watson 지수는 2.13으로 2에 근접하여 잔차의 자기상관 문제도 없음을 확인하였다.

모형 1의 전체 모형은 통계적으로 유의하였으며(F = 5.22, *p* = .008), 설명력은 15.0%로 나타났다. 여성일수록 직무만족도가 낮았으며(β = –.39, *p* = .002), 직무스트레스는 직무만족도에 유의한 영향을 미치지 않았다(β = .14, *p* = .254).

모형 2에서는 생리적 적응 양식인 수면과 신체활동 지표 중 직무만족도와 유의한 상관성을 보였던 총 수면시간(TST)과 일일 걸음 수(daily step count)를 투입하였고, 이와 함께 자아개념 및 역할기능 양식인 전문직 정체성, 상호의존 적응양식인 동료 지지를 모두 투입하였다. 모형 2 역시 유의하였으며(F = 17.53, *p* < .001), 설명력은 65.0%이었다. 회귀모형의 적합성을 확인하기 위해 잔차의 정규성과 등분산성을 검토한 결과, 잔차는 정규분포 가정을 충족하였으며 등분산성도 확인되었다. 구체적인 영향력을 살펴보면, 전문직 정체성이 높을수록(β = .56, *p* < .001), 동료지지가 높을수록(β = .37, *p* < .001), 수면 시간이 길수록(β = .19, *p* = .028) 직무만족도가 높았다.

반면, 일일 걸음 수(daily step count)는 직무만족도에 유의한 영향을 미치지 않았으며, 직무스트레스는 최종 모형에서도 직무만족도에 유의한 영향을 보이지 않았다.

## 논의

본 연구는 로이의 적응이론(Roy's adaptation model)을 기반으로 권역응급의료센터 응급실 간호사의 직무스트레스, 수면 및 신체 활동, 전문직 정체성, 동료 지지가 직무 만족도에 미치는 영향을 통합적으로 파악하고자 시도되었다. 특히, 기존의 단편적인 연구들에서 벗어나 응급실 간호사의 직무 만족도 증진 요인을 적응이론을 기반으로 심도 있게 분석하고, 생리적 적응 양상인 수면과 신체 활동을 액티그래프를 활용하여 객관적으로 측정하였다.

본 연구에 참여한 응급실 간호사의 직무 만족도는 100점 만점 환산시 71.74점으로, 이는 응급실 간호사를 대상으로 한 선행연구[27] 61.50점에 비해 높았다. 선행연구의 대상자는 권역응급센터가 아닌 응급실의 간호사가 38.7%가 포함되었던 점이 이러한 차이를 나타내었을 수 있다. 병원마다 간호사들의 직무 만족도가 상이하게 나타나는 현상은 업무 강도의 차이, 교육시스템 차이, 전문직으로서의 의사결정 자율성의 차이가[28] 있으므로 각 병원규모별 응급의료센터의 특징에 따른 직무 만족도를 확인해 볼 필요가 있겠다.

또한 본 연구에서 응급실 간호사들의 직무스트레스는 100점 만점 71.06점이었다. 이는 응급실 간호사에서 조사된 70.02점[29]과 유사하였으나, 중환자실 간호사에게서 보고된 61.75점[30]에 비해 다소 높은 수준이었다. 이는 응급실이라는 근무환경의 특수성이 반영된 결과로 해석할 수 있다. 응급실 간호사는 환자의 생명과 직결되는 즉각적이고 정확한 업무 수행하며, 동시에 감정 노동, 반복적인 폭력 경험[31], 교대근무로 인한 수면 부족[32] 등 다양한 스트레스 요인에 지속적으로 노출된다. 상대적으로 업무강도가 높은 중환자실 간호사들에 비해서도 응급실 간호사들의 직무스트레스 수준이 높았던 점으로 보아 이러한 복합적 요인들이 작용하여 응급실 간호사들의 직무스트레스 수준을 더욱 가중시킨 것으로 보인다. 따라서 본 연구에서 나타난 응급실 간호사들의 높은 직무스트레스는 단순히 업무량의 과중 때문이 아니라, 응급실 환경의 다차원적, 구조적 특성과 관련되어 있다고 할 수 있다.

그럼에도 불구하고 직무스트레스는 직무만족도와 유의한 상관관계를 보이지 않았는데. 이는 기존의 선행 연구[33]에서 직무스트레스가 직무 만족도에 부정적인 영향을 미친다고 보고된 결과와 상이한 결과이다. 이러한 차이는 다음과 같은 이유로 설명될 수 있다. 첫째, 본 연구는 로이의 적응이론에 기반하여 직무스트레스를 '자극'으로 보고, 스트레스에 대한 다양한 '적응 양식'을 함께 조사하였는데, 직무스트레스 자체가 직무 만족도에 직접적인 영향을 미치기보다는, 직무스트레스에 대한 간호사의 적응 양식이 직무 만족도와 더 관련이 있었을 수 있다. 둘째, 본 연구에 참여한 응급실 간호사들 중 1년 미만의 신규간호사는 4명(6.25%)으로 비교적 적은 편이었으며, 절반 이상은 3년 이상의 임상경력을 보유한 간호사들이었다. 이는 연구에 포함된 대상자들이 이미 응급실 특유의 급박하고 스트레스가 많은 근무 환경에 일정 부분 적응했을 가능성을 시사한다. 실제로 임상경력이 누적될수록 위기 상황 대처 능력과 환자 관리에 대한 자신감이 향상되고, 스트레스 상황을 인지하고 조절하는 전략이 발달한다는 점에서[34] 이들의 직무만족도와 직무스트레스의 연관성이 상대적으로 완화되었을 가능성이 있다. 따라서 응급실 간호사들의 직무 만족도에 대해서 직무스트레스 자체보다는 적응양식이 유의한 영향을 주었던 본 연구의 결과는 적응 여부와 스트레스의 관계에서 적응 양식이 중요한 역할을 한다는 로이의 적응이론이 유효함을 시사한다.

본 연구에서 응급실 간호사들의 수면 특성을 보면, 실제 총 수면시간이 약 6.7시간, 수면 효율은 85.88%, 수면 중 각성 시간이 59.8분으로, 교대 근무자들의 수면 특성[35] 과 유사하였으나, 수면 중 각성 시간이 다소 긴 편이었다. 수면 중 각성 시간은 수면 효율과 직접적인 관련이 있고[36], 인지능력과도 관련이 있는 것으로 알려져 있으므로[37], 정확하고 효율적인 업무 수행을 위해서 응급실 간호사들의 수면 분절을 줄일 수 있는 방안이 필요하겠다. 그러나, 본 연구에서 수면 분절 및 수면 효율은 직무만족도와는 관련을 보이지 않았고 실제 총 수면 시간만이 유의한 관련성을 보여, 직무 만족도에 있어 수면의 질적인 지표 보다는 양적인 지표가 더 관련되는 것으로 나타났다. 이는 선행연구들[38,39]에서 교대근무 간호사의 수면의 질이 낮을수록 직무 만족도가 낮아진다는 연구 결과와 다소 상이하였다. 이러한 이유로는 선행연구들은 수면 특성을 자가보고식 설문지로 측정하여 주관적인 수면의 질을 측정하였으나, 본 연구에서는 액티그래프를 이용하여 객관적으로 수면의 질을 측정하였기 때문일 수 있다. Seo 등[40]은 같은 개념이라도 설문지를 이용하여 주관적으로 측정한 결과와 액티그래프를 이용하여 객관적으로 측정한 결과는 다르게 나타날 수 있다고 보고한 바 있다. 또한 실제 총 수면 시간은 일상생활에서의 활동 능력 및 피로도와 직접적인 관련이 있기 때문에[41], 교대근무 간호사의 경우, 불규칙한 근무 스케줄로 인해 수면의 질적 측면 보다는 확보 가능한 총 수면 시간이 피로 회복에 대한 준비도에 더 큰 영향을 미쳤을 수 있다. 즉, 수면의 질이 다소 저하되더라도 일정량 이상의 수면 시간을 확보하는 것이 직무 만족도에 영향을 주는 중요한 요소로 작용했을 수 있다.

한편, 본 연구에 참여한 응급실 간호사들의 신체활동 특성은 활동대사량 1.37 MET, 일일 걸음 수 12,181.5보, 좌식 활동 641분, 저강도 활동 430분, 중강도 활동 192분으로, 간호사를 대상으로 한 선행연구[42]에서 보고한 8565보에 비해 일일 걸음수가 더 많았다. 신체활동 일일 걸음수는 직무만족도와 유의한 정적 상관관계가 있었으나, 회귀분석에서는 직무만족도와 유의한 관련성을 보이지 않았다. 신체활동과 직무만족도 간의 단순 관련성만 분석한 선행연구들에 의하면 가벼운 신체활동은 직업적 만족감과 상관관계가 있었으며[43], 규칙적인 신체 활동에 참여하는 간호사는 직무 만족도가 더 높은 것으로 나타났다[44]. 신체 활동을 포함하여 직무 만족도에 영향을 미치는 다른 요인들을 통합적으로 분석한 연구가 없어 직접적으로 설명하기는 어렵지만, 추가 분석 결과, 여가·레저 활동 여부에 따른 신체활동 특성을 비교했을 때 유의한 차이가 없었고 여가·레저 활동을 하지 않는 간호사들이 절반 이상으로 신체활동 대부분은 근무 중의 신체 활동으로 추측되는데, 이러한 근무로 인한 신체활동은 다른 요인들과 함께 분석할 경우에 직무 만족도의 큰 영향을 미치지 않았을 수 있겠다.

위계적 회귀분석 결과, 전문직 정체성이 높을수록, 동료 지지가 높을수록, 수면 시간이 길수록 직무 만족도가 증가하였으며, 이 중 전문직 정체성이 직무 만족도에 가장 큰 긍정적인 영향을 미치는 요인으로 나타났다. 전문직 정체성이 높은 간호사는 자신의 실무에서 더 많은 노력과 동기를 보이며, 이는 전문직으로서의 성취감에 기여하고 결과적으로 직무 만족도의 향상으로 이어진다[45]. 따라서 응급실 간호사의 전문직 정체성을 강화하기 위한 교육 프로그램 및 제도적 지원이 직무 만족도 향상에 기여할 수 있을 것으로 보인다.

또한 본 연구에서 동료 지지 또한 직무 만족도에 긍정적인 영향을 미치는 것으로 나타났다. 이는 선행 연구들에서 주로 사회적 지지[46]나 조직의 지원[47]이 직무만족도에 긍정적인 관련이 있는 것으로 보고된 것과 유사한 맥락으로 볼 수 있으나, 동료지지는 사회적 지지나 조직의 지원과는 결이 달라, 선행연구 결과와 다소 다른 측면에서 볼 필요가 있다. 동료 지지는 긍정적인 사회 심리적 업무 환경으로[48], 스트레스 상황에서 회복력을 얻는 데 중요한 요인으로 알려져 있다[49]. 본 연구의 동료 지지는 100점 만점에 71점 정도로 선행 연구[50]의 55점에 비해 높은 편이었는데, 이러한 긍정적인 동료지지는 응급실과 같이 비교적 높은 스트레스 환경에서 특히 중요할 수 있을 것으로 보인다. 따라서 동료 지지는 응급실 간호사들의 직무 만족도를 높이는 데 중요한 역할을 할 수 있으므로, 동료 간 지지를 활성화할 수 있는 조직 문화 조성 및 프로그램 개발이 필요하다.

본 연구의 제한점은 다음과 같다. 첫째, 연구 대상자가 64명으로, 통계적으로 최소 대상자 이상이기는 하나, 상대적으로 적은 수의 권역응급의료센터 간호사들을 대상으로 하였기에 연구 결과를 전체 응급실 간호사 집단으로 일반화하는 데는 제한이 따르며, 일부 변수 간 관계 및 모형의 설명력이 과대추정 되었을 가능성을 배제하기 어렵다. 따라서 향후 연구에서는 보다 큰 표본을 기반으로 모형의 안정성을 확인할 필요가 있다. 둘째, 횡단적 연구 설계로 인해 변수들 간의 인과관계를 명확하게 설명하기 어렵다. 셋째, 직무스트레스 측정 도구가 1984년에 개발된 도구로, 현재의 응급실 환경에서 발생하는 다양한 스트레스 요인들을 충분히 반영하지 못했을 가능성이 있으며, 자가 보고 설문 방법을 이용하여 측정하였기에 응답자의 주관이 개입되어 실제적인 직무스트레스와 차이가 있을 수 있다.

그럼에도 불구하고, 본 연구는 응급실 간호사의 수면과 신체활동을 액티그래프로 측정하여 객관적인 수면 지표와 신체활동 지표를 산출함으로써 수면 분절 및 강도별 신체활동을 기술하였고, 특히 응급실 간호사들에서 수면 분절이 증가되어 있음을 보고하였다는 점에서 그 의의를 찾을 수 있겠다. 또한 응급실 간호사들의 직무 만족도와 관련하여 적응 양상의 중요성을 확인하여, 적응과 스트레스의 관계에서 적응 양상을 강조한 로이의 적응이론이 유효했음을 제시하였다는 점에서 의의가 있다.

## 결론

본 연구는 로이의 적응이론을 기반으로 응급실 간호사의 직무 만족도에 영향을 미치는 다양한 요인들을 통합적으로 분석하고, 특히 액티그래프를 활용하여 수면 및 신체 활동이라는 생리적 적응 양식을 객관적으로 측정했다는 점에서 의의가 있다. 이를 통해 응급실 간호사의 직무 만족도 증진을 위한 다각적인 개입 방안 마련에 필요한 기초 자료를 제공하였다.

본 연구는 응급실 간호사의 직무 만족도에 전문직 정체성, 동료 지지, 그리고 총 수면 시간이 긍정적인 영향을 미친다는 것을 확인하였다. 특히 직무스트레스가 직접적으로 직무 만족도에 유의한 영향을 미치지 않는다는 점은, 간호사의 직무스트레스 자체보다 수면, 전문직 정체성, 동료지지와 같은 적응 관련 요인이 직무만족도와 더 밀접하게 관련될 수 있음을 보여주며, Roy Adaptation Model을 적용한 해석 가능성을 뒷받침한다. 또한 응급실에 근무하는 3교대 근무 간호사의 수면과 신체활동을 액티그래프를 이용해 측정하여 수면 중 각성시간 및 횟수, 강도별 신체활동 시간 등을 객관적인 지표로 제시하고 분석하였다는데 그 의의가 있다.

이러한 연구 결과를 바탕으로 다음과 같은 제언을 하고자 한다. 첫째, 응급실 간호사의 직무 만족도 향상을 위해 전문직 정체성 함양을 위한 교육 및 제도적 지원이 필요하다. 둘째, 동료 간 상호 지지를 증진시킬 수 있는 협력적인 조직 문화 조성 및 프로그램 도입이 요구된다. 셋째, 응급실 간호사의 충분한 수면 시간 확보를 위한 근무 스케줄 조정, 수면 교육 및 상담 지원 등의 노력이 필요하다. 향후 연구에서는 본 연구의 한계점을 보완하여 보다 다양한 지역의 응급실 간호사를 대상으로 종단적인 연구를 수행하고, 최신 직무스트레스 측정 도구를 활용하여 심층적인 분석을 시도할 필요가 있다. 또한, 신체 활동의 질적 측면이나 특정 유형의 신체 활동이 직무 만족도에 미치는 영향에 대한 추가 연구도 제안한다.

## Figures and Tables

**Table 1.** Characteristics of Participants and Job satisfaction (N = 64)

Characteristics	Categories	N (%)	Job Satisfaction	t/F (*p*)
Age (years)	≤ 25	20 (31.2)	118.50 ± 13.14	0.05 (.950)
	26–29	33 (51.6)	117.27 ± 13.22	
	≥ 30	11 (17.2)	117.55 ± 15.44	
Sex	Men	8 (12.5)	130.25 ± 16.10	3.01 (.004)
	Women	56 (87.5)	115.91 ± 12.08	
Education	Technical college	6 (9.4)	117.50 ± 10.67	−0.04 (.969)
	University	58 (90.6)	117.72 ± 13.70	
Living arrangement	With family/friends	32 (50.0)	116.84 ± 10.43	−0.51 (.611)
	Alone	32 (50.0)	118.56 ± 15.92	
Spouse	Yes	9 (14.1)	112.11 ± 8.64	−1.36 (.178)
	No	55 (85.9)	118.62 ± 13.85	
Clinical experience (years)	< 3	31 (48.4)	118.45 ± 15.98	0.69 (.508)
	3–5	16 (25.0)	119.63 ± 8.56	
	> 5	17 (26.6)	114.53 ± 11.90	
Monthly income (million KRW)	3–4	63 (98.4)	117.71 ± 13.49	0.05 (.958)
	> 4	1 (1.6)	117.00 ± 0.00	
Night shift included during study period	Yes	16 (25.0)	118.88 ± 18.64	-0.40 (.689)
	No	48 (75.0)	117.31 ± 11.33	

Values are presented as n (%) or mean ± standard deviation. KRW = Korean Won.

**Table 2.** Descriptive Statistics of Study Variables (N = 64)

Variables	M ± SD	Median (IQR)	Min	Max
Job stress	61.48 ± 8.70	62.50 (9.00)	34.00	79.00
Sleep				
Efficiency (%)	85.88 ± 4.53	86.44 (5.58)	68.04	94.33
TIB (minutes)	465.84 ± 106.29	448.33 (92.00)	311.44	972.33
TST (minutes)	404.43 ± 104.47	374.32 (101.88)	212.78	893.00
WASO (minutes)	59.81 ± 17.91	57.30 (19.80)	28.80	137.43
WE (times)	19.45 ± 5.40	20.24 (7.64)	4.00	32.60
MWE (minutes)	4.07 ± 2.79	3.33 (1.39)	1.93	16.80
Physical activity				
METs	1.37 ± 0.17	1.35 (0.20)	1.11	2.07
Daily step count	9,723.84 ± 2,252.90	9,519.00 (2,736.00)	5,016.00	16,758.00
Sedentary time (min/day)	641.34 ± 124.30	664.29 (121.07)	231.43	910.71
Light activity time (min/day)	430.48 ± 76.08	454.29 (91.61)	145.71	565.71
Moderate activity time (min/day)	192.13 ± 47.36	188.57 (58.93)	64.29	304.29
Vigorous activity time (min/day)	0.00 ± 0.00	0.00 (0.00)	0.00	0.00
Professional identity	63.70 ± 6.77	63.50 (9.00)	45.00	80.00
Peer support	32.61 ± 3.97	32.50 (6.00)	22.00	40.00
Job satisfaction	117.70 ± 13.38	117.00 (19.00)	89.00	161.00

M = Mean; SD = Standard deviation; IQR = Interquartile range; Min = Minimum; Max = Maximum; TIB = Time in bed; TST = Total sleep time; WASO = Wake after sleep onset; WE = Wake episode; MWE = Mean wake episode; METs = Metabolic equivalents of task.

**Table 3.** Correlation between Study Variables (N = 64)

Variables	LN job stress	Professional identity	LN peer support	LN TST	LN sleep efficiency	Moderate physical activity	Daily step count	Job satisfaction
	r (*p*)							
Professional identity	−.05 (.705)	1	-	-	-	-	-	-
LN peer support	−.04 (.746)	.25 (.049)*	1	-	-	-	-	-
LN TST	−.10 (.429)	.07 (.588)	.16 (.211)	1	-	-	-	-
LN sleep efficiency	−.01 (.934)	.05 (.677)	.06 (.660)	.56 (< .001)***	1	-	-	-
Moderate physical activity	−.05 (.719)	.13 (.321)	.28 (.023)*	−.12 (.365)	−.04 (.767)	1	-	-
Daily step count	−.07 (.600)	.20 (.113)	.29 (.022)*	.11 (.374)	.08 (.536)	.69 (< .001)***	1	-
Job satisfaction	.04 (.751)	.67 (< .001)***	.55 (< .001)***	.28 (.024)*	.05 (.672)	.24 (.059)	.27 (.032)*	1

LN = Natural log-transformed; TST = Total sleep time. *p < .050; ***p < .001.

**Table 4.** Factors Affecting Job Satisfaction (N = 64)

Variables	Step 1					Step 2				
	B	SE	β	t	*p*	B	SE	β	t	*p*
(constant)	97.33	41.96		2.32	.024	−190.92	53.08		−3.58	.001
Sex (women) (ref.: men)	−15.79	4.91	−.39	−3.21	.002	−0.69	3.80	−.02	−0.18	.856
LN job stress	12.17	10.56	.14	1.15	.254	9.34	7.05	.11	1.33	.190
LN TST						11.04	4.91	.19	2.25	.028
Daily step count						0.00	0.00	.04	0.44	.661
Professional identity						1.10	0.17	.56	6.64	< .001
LN peer support						38.35	9.38	.37	4.09	< .001
R^2^	.15					.65				
adj R^2^	.12					.61				
F	5.22					17.53				
*p*	.008					< .001				

ref. = Reference; LN = Natural log-transformed; SE = Standard error; TST = Total sleep time.
